# The Influence Mechanism of Political Skill on Safety Voice Behavior in High-Risk Industries: The Mediating Role of Voice Efficacy

**DOI:** 10.3390/ijerph192316162

**Published:** 2022-12-02

**Authors:** Yunfeng Sun, Jianwu Chen, Chongyang Qian, Xiaowei Luo, Xiang Wu

**Affiliations:** 1 School of Engineering and Technology, China University of Geosciences (Beijing), Beijing 100083, China; 2 Institute of Occupational Hazards, China Academy of Safety Science and Technology, Beijing 100012, China; 3 Institute of Urban Safety and Environmental Science, Beijing Academy of Science and Technology, Beijing 100054, China; 4 Department of Architecture and Civil Engineering, City University of Hong Kong, Hong Kong 999077, China

**Keywords:** political skill, safety voice behavior, voice efficacy, mediation

## Abstract

As an important indicator to measure the adaptability and development potential of individuals in an organization, political skill is rarely considered as an antecedent variable in the field of safety voice. This study is based on impression management theory and social cognitive theory. From the perspective of employee self-service-oriented safety voice motivation, we took political skill as a predictor of safety voice behavior and introduced voice efficacy as an intermediary variable to construct a theoretical model of the relationship between political skill, voice efficacy, and safety voice behavior. We used the method of questionnaire to collect data from employees in high-risk industries. SPSS and AMOS software were used as analysis tools to examine the relationship between political skill, voice efficacy, and safety voice behavior. The study results show that: (1) political skill has a significant positive impact on safety voice behavior; and (2) voice efficacy plays a mediating role in the relationship between political skill and safety voice behavior. In this study, a new predictor of safety voice behavior and its mediation mechanism were obtained. Political skill can not only reflect the psychological cognitive ability of individuals in dangerous work environments, but it is also an important manifestation of the social exchanges between employees and organizations. In the special organizational context where China generally values “guanxi”, political skill has a stronger ability to predict and explain safety voice behavior. This research can help organizations obtain safety-related suggestions from employees in a timely manner and realize the sustainable development of safety management.

## 1. Introduction

With the increasing number of accidents caused by unsafe behaviors, organizations are becoming more aware of the impact of human factors. A large number of human-oriented safety management studies have begun to emerge. Among them, employee safety voice behavior has caused more and more attention [1,2,3,4,5]. If individuals conceal safety-related issues of the organization and do not report hidden dangers they find, it may increase the difficulty of risk prediction and prevention, which will make the risk impossible to control and lead to safety accidents [6,7]. Safety voice means expressing personal opinions and inner concerns on workplace safety issues [8]. The positive effect of safety voice on the organization’s safety management work has been verified in corporate practice. Previous studies have also shown that safety voice can help improve the organization’s production safety status [4,8,9,10]. Safety voice is a breakthrough for organizations to realize the sustainable development of safety management [3,11]. However, due to the nature of safety voice behavior, the actual dilemma of employees implementing safety voice behavior urgently requires continued research.

Safety voice is the act of expressing opinions on safety issues in the workplace, and safety voice is generally directed at leaders [5,12,13]. As an extra-role behavior, safety voice may involve risk control and hidden danger prevention, along with high interpersonal risks [14]. Safety voice is a behavior outside the scope of responsibility requirements, which means that safety voice behavior requires employees to disperse their corresponding work resources and energy, and they also need to face unknown safety voice results. Therefore, the intention of employees to provide safety voice to their superiors or colleagues is often low [15]. Speaking out about safety in the workplace is a behavior with high interpersonal risk, and the range of psychological activities, such as mental modeling and risk assessment, that individuals undertake prior to committing a constructive act, can be more sensitive to these potential social rules [11,14]. Political skill, as an important criterion for measuring the level of interpersonal relations, reflects the ability of employees to manage relationships with colleagues and leaders [16]. Individuals with higher levels of political skill will have greater confidence in controlling interpersonal risks. They know how to speak out about safety issues without arousing resentment from others. Research showed that political skill motivate employees to voice their opinions [17]. Most of the previous literature on safety voice has focused on individual-level psychological perceptions and organizational-level situational factors, and most of it is based on Western contexts [4,15]. Yet, individuals’ cognitive habits and psychological perceptions in organizations are often also influenced by specific geographical and cultural differences [18]. The specific social context in China determines the importance of political factors in organizations. Still, little research has focused on how political skill influences employee safety behavior in Chinese organizational contexts.

A high level of political skill means strong social influence ability, a good social network, and a wide range of information acquisition channels [19]. Therefore, employees with strong political skill usually have richer psychological capital to strengthen their belief in completing voice behaviors and receiving good feedback, that is, a higher level of self-efficacy [20]. In the context of the specific research on safety voice, we can call it the voice efficacy. The organizational behavior of employees is often closely related to the political atmosphere of the organization. Interpersonal relationships in Chinese organizations often affect the promotion and development of employees in the organization to a certain extent. Therefore, employees usually tend to manage the relationship with their leaders and colleagues as their capital in the organization [21]. They will judge whether their suggestions have disrupted interpersonal harmony or challenged leadership authority and, finally, decide whether to implement voice.

Self-efficacy is an individual’s judgment of his or her ability to accomplish a task [22]. Past research has confirmed that self-efficacy motivates employees to speak out about their work and can mediate the relationship between leadership behavior and employee voice [23,24]. As a special type of efficacy, voice efficacy refers to individuals’ deep cognitive beliefs about their perceptions of competence in the role of a proponent and good expectations of the effectiveness of the proponent [25]. However, scholars have mainly focused on the mechanisms of self-efficacy on employee behavior, and few studies have addressed the role of voice efficacy on voice. Although a few studies have verified the positive effect of voice efficacy on employee voice, they have not addressed the safety domain, i.e., safety voice [26,27,28]. We proposed that political skill has a positive relationship with voice efficacy. Employees with high political skill have the ability to build rapport with others in the organization [16,29], and they perceive that, even if safety voice is challenging, it will not be resented by others. In addition, employees with high voice efficacy believe that they are capable of participating in safety voice and are recognized by their leaders, thus actively participating in safety voice behavior. Therefore, this study introduced voice efficacy as a mediator between political skill and safety voice and explored the role of voice efficacy on safety voice to complement antecedent research on safety voice.

“Guanxi” refers to interpersonal relationships based on specific criteria, formed when both parties within an interaction provide resources to support each other [30]. “Gianxi” has a significant role in Chinese society and organizations [31,32]. Considering that Chinese society generally values the “guanxi” characteristic, this study is based on impression management theory and social cognitive theory. We introduce political skill, a new variable closely related to “guanxi”, and we interpreted the influencing factors and mechanisms of safety voice from the perspective of social interaction. In addition, we used voice efficacy as an intermediary variable to construct the model framework of this study. This study attempts to explore the relationship between political skill, voice efficacy, and safety voice. At the same time, it can provide a new idea and practical basis for organizations to motivate employees to make safety voice.

Overall, this study will contribute to the existing literature in four ways: first, by empirically examining the relationship between political skill and safety voice, it responds to the academic call to explore the antecedent variables of safety voice from the perspective of employees’ individual competencies; second, by examining the mediating role of voice efficacy, it reveals how political skill motivate safety voice, thus opening the black box of the positive effects of political skill; then, the model of the role of political skill constructed based on impression management theory deepens the understanding of the positive consequences of political skill in the workplace, thus deepening the academic understanding of the issues in this area; finally, the drivers of employee safety voice in high-risk industries are explored to provide input for improving safety in high-risk industries.

## 2. The Literature Review and Research Hypothesis

### 2.1. Safety Voice

The concept of safety voice originated from the research of employee voice, aiming to boldly express opinions or concerns related to workplace safety issues [12,33]. Voice and safety voice all include extra-role behaviors to solve and perfect organizational deficiencies [14,34]. Early research on safety voice is closely related to concepts such as safety participation [35], safety citizenship behavior [36,37], proactive behavior [38,39], and pro-social safety behavior [38,39]. With the deepening of research, scholars gradually discovered the features of safety voice from other organizational behaviors and separated them from the above similar concepts. First, the research background of safety voice mainly considers organizations in dangerous work environments. Safety-related behavior has become the main manifestation of the connotation of safety voice, which can be considered as a special type of employee voice. Second, safety voice highlights the willingness of employees to take the initiative. Individuals, for the purpose of their own career or organizational safety and sustainable development, put forward their own constructive opinions on safety issues that have not been considered in the organization [7], which exceeds the scope of the organization’s existing safety regulations and programs. Third, the current research on safety voice focuses more on combining research results in areas such as safety culture, safety atmosphere, and psychological safety [40]. It emphasizes the unique safety voice factors, such as safety environment, autonomous motivation, and interpersonal risk [33]. Its particularity is also manifested in the level of the content of the voice, the logic of occurrence, the way of voicing, the recipient, the mechanism of the antecedent variables, and the effect variables. Current scholars have inconsistent views on the concept of safety voice. For example, Bienefeld and Grote [41] argued that safety voice is the act of speaking out boldly to prevent bodily harm in dangerous situations. Curcuruto and Griffin [42] noted that safety voice is a form of proactive safety behavior that goes beyond safety regulations. However, from the perspective of organizational behavior, most scholars still agree that safety voice is firstly driven by the initiative and constructive purpose of the individual. The employees then verbally convey suggestions on safety-related issues to colleagues or superiors to change the status quo, including obvious extra-role behaviors [8,38,43].

Scholars have systematically and integrally studied safety voice from the levels of individual and situational factors, driving mechanisms, investigation methods, and the essence of conceptual connotation. However, the integrated research on safety voice is still developing slowly at this stage [44]. The research on the antecedent variables and mechanisms of safety voice needs to be explored and expanded. Therefore, based on the context of Chinese localized organizations, we introduced the concept of political skill, which seems to have a distant relationship with safety voice as the antecedent variable, and we examined the mediating role of voice efficacy. In addition, this research starts from the employees’ characteristics and psychological cognition and takes interpersonal social interaction as an entry point to deeply explore the driving mechanism of safety voice.

### 2.2. Political Skill

In the field of organizational behavior research, it is critical to consider individual political factors. The importance of political factors to organizations has been recognized by many scholars, and the political strategies adopted by employees in organizations have long been valued by researchers [45]. Political skill level can not only determine whether an individual can quickly adapt to the organizational environment, but it is also an essential factor affecting individual career development. Unlike other social effectiveness indicators (such as profit per capita, labor productivity, and operating income), political skill is often used in work scenarios [46]. As a special kind of personal ability, political skill level can be used as an indicator to measure the individual’s interpersonal communication ability, and, to a certain extent, reflects the individual’s interpersonal influence in the organization. By playing the active role of political skill, individuals can achieve professional development and personal success [47]. Perrewé et al. [48] examined that individua ls with strong political skill are good at adjusting the relationship between role overload and work stress, and they effectively reduce its negative effects. Political skill can also better influence others to achieve personal goals, giving employees certain advantages in job promotion and career success [49]. Ashforth et al. [50] pointed out that leaders’ political skill can positively affect team performance. Wei et al. [21] also found that political skill contributes to the development of employees and the effective use of organizational resources by individuals. Xue et al. [17] focused on the individual level, using political skill as a predictor, explored its positive effects on employee voice behavior, and verified the mediating role of impression management motivation in the model.

Safety voice is closely related to organizational citizenship behavior and proactive behavior. Political skill has been introduced by many scholars into the study of employee spontaneous organizational citizenship behavior and employee initiative behavior, and it has been empirically proven in theory and practice. However, the effects of political skill on safety voice behavior in the context of safety-critical organizations are still lacking. Therefore, whether political skill affects and how it affects employee safety voice still need relevant research evidence.

### 2.3. Voice Efficacy

The construct of voice efficacy is derived from the study of self-efficacy, which is the individual’s confidence in being competent for the role of voicer and gaining the ideal effect of voice [51]. Self-efficacy is an assessment of a person’s confidence and belief in their capacity to accomplish their goals [52], while the voice efficacy is a special form of self-efficacy in a specific organizational context. Detert and Burris [53] found that higher performance improves employees’ sense of self-efficacy, so high-performing employees are more inclined to express their opinions than low-performing employees. Self-efficacy has a positive effect on people’s psychological cognition and behavioral decision-making. Tierney and Farmer [54] pointed out that work self-efficacy can affect the creative performance of employees by affecting creative self-efficacy. Therefore, we have reason to believe that self-efficacy can influence voice behavior through the mediating effect of voice efficacy. The role of self-efficacy can not only be reflected in general situations, but also in specific tasks or situations. Gist [22] argued that, since self-efficacy in a special context is more closely related to people’s cognition and behavior, the efficacy of a specific field has a stronger predictive power for corresponding behavior. Previous studies have shown that employees’ voice efficacy can predict their voice behavior [55]. The strength of confidence in obtaining the desired effect of voice directly affects employees’ voice intentions. Therefore, voice efficacy may contribute to safety voice.

As self-efficacy for specific individual behavior, the predictive effect of voice efficacy on employee voice behavior has been verified and supported by previous studies. However, it remains to be explored whether or not voice efficacy can have the same effect on safety voice behavior in specific situations. The decision-making process of the individual before the output behavior determines whether the individual will take actual actions. As a source of belief for employees to insist on voice behavior and gain positive results, voice efficacy is a key link in making voice behavior. Therefore, analyzing and investigating the relationship between voice efficacy and employee safety voice behavior is helpful in enriching the research framework of safety voice.

### 2.4. Research Hypothesis

#### 2.4.1. Employees’ Political Skill and Safety Voice Behavior

Similar to the research on employee voice, most of the existing voice-related literature tends to adopt social exchange theory. These studies believe that, to better serve the organization and colleagues, employees conduct social exchanges with the organization in the form of voice on the premise of mutual benefit, that is, altruistic factors are the main motivation for the voice behavior. However, Ng and Feldman [56] argued that previous studies had neglected the possibility that employees can adjust their own resources for self-service motives by using voice behavior. Van Dyne et al. [43] also emphasized that the motivational tendency of voice behavior includes both being other-oriented and self-oriented. The research results of Detert and Edmondson [57] on employees’ implicit beliefs in voice also showed that voice behavior is influenced by organizational political factors because employees will evaluate the feasibility of voice behavior to obtain more resources for career development. Based on this perspective, this research focused on self-service motivated safety voice behavior by discussing its relationship with political skill.

Employees usually use safety voice with their leaders through informal channels based on language communication. This process is often accompanied by challenges to the status quo and the risk of interpersonal conflict. Under the Chinese cultural background, most organizations have the characteristics of high power distance and political atmosphere. To win limited resources, political strategies are widely used in Chinese organizations [58]. If employees want to leave a good impression on their leaders and colleagues, they first need to have a sincere attitude to understand others during their interactions with members of the organization to establish a good interpersonal network. Then, they use this to influence one’s image in the hearts of others to achieve the set goals. Employees with high political skill can influence others through interpersonal strategies, such as persuasion, probation, and control, to avoid or reduce the negative impact that voice may have on themselves [16].

Goffman [59] noted that impression management is an attempt to influence others’ impressions of themselves. With the deepening of research, impression management theory has been introduced into the field of organizational behavior by more and more scholars. It has become a critical theory to explain individual motivation and behavior. Tetlock and Manstead [60] divided impression management into acquired impression management and protective impression management from the perspective of the function and role expected by the individual. Fuller et al. [61] argued that voice can be regarded as a strategy to enhance one’s impression in the organization. The facilitative voice proposed by Liang et al. [62] also fits with the impression management theory at the level of the individual’s psychological motivation and behavioral tendency. According to impression management theory, employees in the workplace are eager to establish their desired image in the organization. This is because companies need and value employees who do a good job and will offer them rewards or promotions. To improve their careers and workplace safety, employees adopt acquired impression management strategies and put forward safety recommendations to the organization to gain support and appreciation from leaders and colleagues. When faced with risky events, individuals will also adopt protective impression management strategies to reduce the negative impact of risk events on themselves and prevent others from having negative impressions. Employees in the organization keep silent on safety issues because they often avoid interpersonal conflicts to maintain a positive image in the minds of leaders. In the same way, the reason why employees remain silent on safety issues in the organization is to avoid interpersonal conflicts to maintain their positive image in the hearts of leaders. Therefore, for individuals with high motivation for impression management, they pay more attention to the external evaluation of their image and behavior. In addition, they pay more attention to the inner evaluation formed by self-judgment. This pursuit of external evaluation and internal cognition usually drives them to make behaviors conducive to establishing a positive image. Driven by the motivation of impression management, individuals will deliberately or unintentionally develop and improve the ability to obtain a good evaluation from the outside world and build a good impression in others, that is, the formation and improvement of political skill. As the behavior that may promote the organization’s development and bring better performance evaluation to the individual, voice behavior is an important manifestation of the use of political skill to regulate resources [47,63,64,65]. Therefore, employees with impression management motives tend to be more likely to possess the corresponding political skill, and further form a higher initiative to make voice. In addition, voice behavior can be used to reflect work ability and attitude. Individuals with high motivation for impression management expect to leave a better impression on the organization, so they are more inclined to raise workplace safety issues. In the context of an organization, employees tend to achieve their own impression management goals by actively providing advice and suggestions to the organization. For employees with a higher level of political skill, using safety voice as a specific form of implementing an impression management strategy can achieve better results. Therefore, they are more inclined to adopt appropriate methods to put forward suggestions on safety issues to gain the recognition and attention of the organization. Specifically, when facing safety issues in the workplace, employees with higher levels of political skill can choose more appropriate methods and opportunities to present their views on safety issues to their leaders and colleagues. They tend to have more precise control over when, where, and how to make a suggestion. Therefore, their safety voice behavior is more conducive to establishing an image of caring about the organization’s production activities, and it is more conducive to making the organization feel that they are integrated into the collective and the enthusiasm of serving the collective so as to obtain good feedback from the object of the voice. At the same time, employees with high political skill will strengthen the positive impact of safety voice on themselves by showing sincerity in the process of safety voice, and better hide their motivation to please the organization and avoid its negative impact [47,66]. In summary, employees with strong political skill can play the role of impression management of safe voice behavior. In the daily work process, they are more inclined to actively provide safety voice to leave a good impression on others.

Therefore, this research proposes that employees with higher levels of political skill have more safety voice behavior and are more inclined to propose organizational safety issues. Based on this, we hypothesize:

**H1**  :*Political skill has a significant positive impact on employee safety voice behavior.*

#### 2.4.2. The Mediating Role of Voice Efficacy

A high level of political skill means that individuals can have considerable network resources, and, at the same time, build a good image in front of other members of the organization, which makes the suggestions of voicers widely valued by members of the organization [67]. Additionally, the proper use of political skill can regulate the relationship between employees, colleagues, and bosses, and reduce the risk of interpersonal conflict and negative consequences that may be brought about by the voice behavior. Then employees’ belief in voice has been improved [55]. According to previous studies, employee safety voice behavior is often based on informal channels such as verbal communication [10]. Individuals with a higher level of political skill can choose a good object, timing, occasion, technique, and form of voice based on specific circumstances. This ability will positively affect the individual’s voice efficacy and enhance employees’ belief incompetent voice behavior.

**H2:**  *Political skill has a significant positive impact on voice efficacy.*

According to social cognition theory, the environment, people, and behavior constitute a dynamic mutual determination relationship, which plays a key role in shaping individual behavior [68]. It emphasizes the importance of self-efficacy as an individual cognitive factor [52]. With the development of research, the social cognitive theory has been widely used to understand and predict the behavior and characteristics of individuals and groups. The theory proposes that self-efficacy can more directly affect individual behavior than other cognitive factors [69]. Compared with the general sense of self-efficacy, the distance between the sense of efficacy in a specific field and the behavior in that field is closer, and the relationship between the two is often closer [22]. Therefore, we conclude that voice efficacy is a closer construct to voice behavior, which can well reflect the degree of self-confidence and motivation of individuals who tend to speak on safety issues.

From the perspective of voice efficacy on safety voice behavior, improving voice belief and the stability of motivation are particularly critical. Tangirala and Ramanujam [70] focused on the internal motivation of the individual. They argued that a person’s attitude could predict his voice behavior, and the voice behavior is an employee’s positive behavior towards work. Kish-Gephart et al. [51] pointed out that the formation of voice efficacy is mainly derived from the individual’s own experience of voice behavior. As one of the core constructs of social cognitive theory, general self-efficacy is a domain- and situation-independent measure of people’s confidence in what they can achieve, and it is an important predictor of behavior [27]. When voice efficacy is high, people feel empowered to speak up about safety and expect better outcomes; conversely, when voice efficacy is low, people feel powerless, easily indifferent, and remain silent even when safety issues are identified. Individuals with strong self-efficacy tend to gain confidence from their successful experiences in other fields to improve their level of voice efficacy. Before making safety voice, individuals will evaluate and analyze issues, such as whether to provide safety voice and the difficulty of controlling the voice process based on their conditions and experience. Individuals with a high sense of self-efficacy often make lower evaluations of the difficulty and risk of the voice behavior. They have higher expectations for the possibility of the voice to be adopted and the effect of the voice, so they tend to make voice, regarding the psychological perception and assessment of the risk of voice. Curcuruto et al. [71] divided safety voice into two stages: psychological simulation and the implementation of advice behavior. They also pointed out that individuals often conduct psychological rehearsal of the implementation process and interpersonal risks before issuing safety recommendations to their superiors to evaluate the feasibility of the voice behavior. In terms of the expectation of the effect of voice, Van Dyne and LePine [72] found that voice behavior affects the leadership’s evaluation of employee performance. Whiting et al. [73] also examined that the evaluation of employees by leaders will be affected by employees’ voice behaviors. It is not difficult to find that reasonable safety voice behavior can make employees leave a positive impression on leaders, which is closely related to their career success, and which is also a driving force for employees to implement voice behavior. Additionally, a higher level of voice efficacy also determines that individuals can invest more time and energy in the process of voice behavior and have higher psychological resilience. This allows individuals with high voice efficacy to persist in their voice behaviors. They will also have stronger confidence that their suggestions can ultimately achieve the desired effect of voice. Based on the above analysis, this research proposes the following hypotheses:

**H3:**  *Voice efficacy has a significant positive impact on employee safety voice behavior.*

**H4:**  *Voice efficacy plays a mediating role in the relationship between political skill and safety voice behavior.*

**Figure 1:**
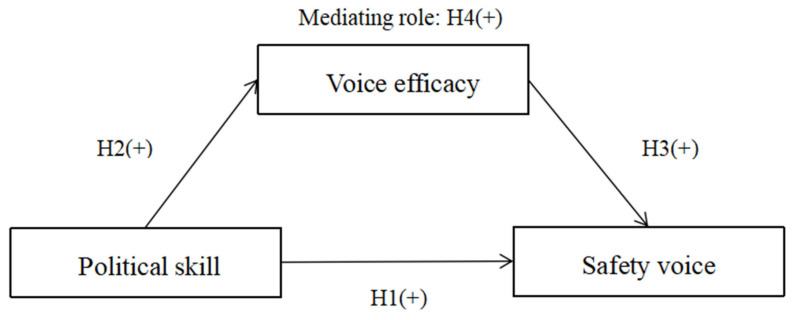
Research hypothesis model.

In summary, we hypothesize that political skill can predict employee safety voice behavior through the mediating role of voice efficacy. Thus, we construct the research hypothesis model for this study (shown in Figure 1).

## 3. Method

### 3.1. Research Objects and Procedures

High-risk industries often cause accidents due to failure to address safety issues in a timely manner, and these accidents can result in significant casualties [4,74,75]. Safety voice from high-risk industries is extraordinarily important, so we selected workers in high-risk industries as survey respondents. This study selected employees in high-risk industries, such as construction and mining in China, as the research sample. Considering that safety voice belongs to the category of extra-role behaviors, the selection of samples did not involve employees involved in on-site safety management, such as safety officers and safety managers. When designing the research program, the following measures were mainly taken to avoid serious homology variance problems. To prevent the respondents from not filling in the answers according to their true inner thoughts, the respondents were told that the questionnaire was an anonymous survey before answering the questionnaire. Moreover, to avoid deviations in the subject’s understanding of the items, and in compiling the questionnaire, the expression of the question was improved to make it as easy to understand as possible.

The questionnaire was divided into two parts. The first part was the investigation of basic information, including gender, age, education level, working years, and position. The second part involved the variables studied in this paper, namely, political skill, voice efficacy, and safety voice. All the scales measuring the three variables are mature scales. The questionnaires in this study were generated through the Questionnaire Star platform, and the researcher sent questionnaires via WeChat to human resource leaders involved in high-risk industries, such as construction and hazardous chemicals. These leaders sent questionnaires to work WeChat groups, which were filled out by workers. The intention of the study was explained to the subjects, and their verbal consent was obtained before filling it out.

We set options in the questionnaire to ask whether the respondents were safety managers and workers in high-risk industries, and we removed the questionnaires completed by safety managers and workers in high-risk industries from the questionnaires received. In addition, we identified those questionnaires that were completed in a short time and those with concentrated answers as invalid. We received a total of 361 questionnaires, and we obtained 245 valid questionnaires after screening.

We used SPSS to conduct descriptive analysis, reliability analysis, correlation analysis, and hierarchical regression analysis on the questionnaire, and we used confirmatory factor analysis using AMOS.

### 3.2. Measuring Tools

The variables to be measured in this study are: political skill, voice efficacy, and safety voice behavior. To ensure the reliability and validity of various variable measurement tools, the measurement tools used in this study are all widely recognized mature scales. All scales in the questionnaire use a 5-point scoring method, with one indicating “completely non-conformance” and five indicating “completely conforming”.

#### 3.2.1. Political Skill

The measurement of political skill used a scale compiled by Ferris et al. [76], containing 18 items. Measurement items were those such as “When communicating with other people, I try to make my words and deeds appear sincere”, “I try to show that others are interested in his/her affairs, and “It is essential to make others think that I am sincere in speaking and doing things”. Ferris et al. [76] developed a measurement tool for political skill based on their previous research combined with research results in related fields. Their research divided political skill into four dimensions: social alertness, interpersonal influence, network ability, and apparent sincerity. The scale has been used extensively in previous studies, and it has shown good reliability and validity in empirical research. Although the scale is derived from Western research, some scholars have found it to be an equally accurate measure in the Chinese cultural context [58]. Therefore, the study chose this scale as a measurement tool for predictors.

#### 3.2.2. Voice Efficacy

We used the scale compiled by Duan and Wei [28] to measure voice efficacy, containing seven items. Measurement items were those such as “I can effectively control the impact of the incident on me when advising to the company when encountering an emergency”, “No matter what occasion, I can express my reasonable suggestions on the affairs of the company”, and “My opinion can attract the attention of leaders”. The scale compilation is based on qualitative research ideas concerning previous relevant research concepts, and it completed the construction of the structure and the compilation of the scale with Chinese culture as the background. Considering the differences in social culture and organizational systems, the scale compiled under local culture is more suitable for research on Chinese organizations. At the same time, after the scale was proposed, scholars introduced it into the research to test its credibility and validity of the scale. In short, considering the good applicability of the scale to the research object and its high degree of recognition, the study decided to use the scale as a measurement tool for intermediate variables.

#### 3.2.3. Safety Voice Behavior

In this study, a 5-item scale developed by Tucker et al. [12] was used to measure safety voice behavior. Measurement items were those such as “I can make suggestions to improve work safety, “When I find colleagues doing unsafe things, I will stop them”, and “I will discuss new ways to improve workplace safety with colleagues or leaders”. Since the scale was proposed, it has been adopted by many scholars, and the results have shown good reliability and validity. However, applying this scale to research in the local context of China is still relatively rare. In the empirical study of the scale, the subjects are grassroots employees, which is consistent with the job level of most survey subjects in this study. This consistency helps the scale to show higher validity in this study. Additionally, the results of the reliability and validity analysis of the survey data show that the scale has a high degree of reliability and shows good validity. Therefore, the study uses this scale to measure variables.

#### 3.2.4. Control Variables

Previous studies found that individual factors such as gender [77] and age [2] can affect the intention of safety voice. Therefore, this study introduces five demographic variables: gender, age, education level, working years, and position as control variables to avoid interference with the result analysis.

### 3.3. Data Processing

Existing studies have found that demographic variables may impact safety voice [72]. To understand the basic structure of the sample, this study conducted a frequency analysis on the gender, age, education level, working years, and job level of the subjects. To test the consistency of the data prediction level, this study used the Cronbach coefficient to test the reliability of the various scales involved in the study. At the same time, to test how close the predicted level of the data is to the actual class, this study conducted a validity analysis of the variables. Considering that the scales used in the study are all derived from the research of well-known scholars and have shown good validity in the follow-up research, the study mainly carried out a discriminative validity analysis on variables. In addition, this study conducted correlation analysis and regression analysis to better explain the relationship between the various variables.

## 4. Results

### 4.1. Sample Descriptive Statistics

This survey recovered 361 questionnaires, and 245 valid questionnaires were obtained after screening, with an effective rate of 67.9%. It can be seen from the statistical analysis that there are more males in the subjects (the proportion of males is 72.2%, and the proportion of females is 27.8%). The subjects’ ages were mainly 25–34 years old and 35–44 years old. Proportions were 44.9% and 26.5%, respectively. The proportions of subjects under 25 and over 45 were 15.5% and 13.1%, respectively. In terms of education level, the proportion of Bachelor’s degree holders is 53.1%, the proportion of college degree and below is 29.8%, and the proportion of Master’s degree and above is 17.1%. In terms of working years, subjects with six to 10 years of service and more than ten years accounted for a larger proportion, 49.4% and 33.5%, respectively, and subjects with one to five of service accounted for 14.7% of the total number. The number of subjects with a working experience of less than one year was relatively small; the proportion was 2.4%. From the perspective of job level, the subjects participating in the questionnaire survey are mainly ordinary employees; the proportion is 55.1%. Grassroots managers and middle and senior managers accounted for 33.9% and 11.0% of the total number, respectively.

### 4.2. Reliability and Validity Analysis

#### 4.2.1. Reliability Analysis of the Scale

The reliability test results of the scale are shown in Table 1. The results show that the Cronbach coefficient of each subscale is greater than 0.7, indicating that the scales used in this study have good consistency and stability.

#### 4.2.2. Confirmatory Factor Analysis

We performed confirmatory factor analysis using AMOS to test the validity of the model. The study compared single-factor, two-factor, and three-factor models to test the discriminative validity of each variable. The test results are shown in Table 2. The results show that, compared with single-factor and two-factor models, if the measurement model is represented by a three-factor model, all data indicators can meet the standard requirements. The analysis results can show better goodness of fit ($\chi^{2}/df$ = 1.675, RMR = 0.033, RMSEA = 0.053, CFI = 0.909, and IFI = 0.910). Therefore, the variables in the study have good discrimination validity.

### 4.3. Analysis of Common Methods Variance

We used the Harman single factor test and common latent factor to test for common bias. Before the Harman single factor test, it was necessary to test whether the research data was suitable for factor analysis. The study used SPSS software to analyze the data and found that the KMO value was 0.938. The Bartlett spherical test value was significant (*p* < 0.01), making it suitable for factor analysis. Without rotation, through exploratory factor analysis, the number of factors with feature roots greater than one extracted is 4 (more than 1). The factor with the most explanatory power can explain 37.147% (less than 40%) of the total variation. Therefore, there is no common method variance problem in this study. In addition, we used the common latent factor and added the common method variance factor to the three-factor measurement model, and the model fit was fair ($\chi^{2}/df$ = 1.589, RMR = 0.035, RMSEA = 0.049, CFI = 0.912, IFI = 0.923). The small change in each fit index indicated that the model, with the addition of the common method variables, did not significantly improve the model. Therefore, there is no significant common method bias.

### 4.4. Correlation Analysis

The study conducted a correlation analysis on each variable, and the results are shown in Table 3. The analysis results showed that political skill had a significant positive correlation with safety voice behaviors (r = 0.56, *p* < 0.01) and a significant positive correlation with voice efficacy (r = 0.74, *p* < 0.01). There was a significant positive correlation between voice efficacy and safety voice behavior (r = 0.66, *p* < 0.01). By analyzing the results, it is not difficult to see that the antecedent variables, intermediate variables, and outcome variables involved in the study show a pairwise correlation. Therefore, the variable relationship of the research hypothesis has been initially verified.

### 4.5. Hypothesis Testing

We used a hierarchical regression method. We controlled for demographic variables such as gender, age, and education. The study adopted the regression analysis method, using political skill as a predictor variable and safety voice behavior as a predicted variable and introduced it into the regression equation. t-values with “*” indicated *p* < 0.05, and with “**” indicated *p* < 0.01, i.e., significant results. Because the regression analysis mainly verified the relationship between political skills, voice efficacy, and safety voice, we did not remove descriptive variables when they were not significant. The analysis results are shown in Table 4, indicating that political skill has a significant predictive effect on safety voice behavior (B = 0.70, t = 10.31, *p* < 0.01). Therefore, Hypothesis 1 is verified.

To validate the mediating role of voice efficacy in the theoretical model, the data analysis steps of the study were as follows: firstly, the study conducted regression analysis with political skill as the predictor variable and voice efficacy as the variable being predicted. As shown in Table 4, the positive predictive effect of political skill on voice efficacy was significant (B = 0.76, t = 17.08, *p* < 0.01), and the analysis results satisfy Hypothesis 2. The study then conducted a regression analysis with political skill and voice efficacy as predictor variables and safety voice behavior as the predicted variable. The results are shown in Table 4. The positive predictive effect of voice efficacy on safety voice behavior was significant (B = 0.68, t = 7.60, *p* < 0.01), and the analysis meets Hypothesis 3.

Finally, the bootstrap method was used to test the mediation effect, and the results are shown in Table 5. Both the upper and lower limits of the 95% confidence interval of bootstrap = 5000 do not contain 0 (the lower limit is 0.34, and the upper limit is 0.70), indicating that voice efficacy plays a positive intermediary role between political skill and safety voice behavior. Therefore, Hypothesis 4 is finally verified.

## 5. Discussion

What are the factors that affect employee safety voice behavior? Some scholars argued that factors such as age [2], gender [77], perception of organizational safety support [12], safety improvement idea [8], and safety transformational leadership [37] could influence safety voice behavior. In terms of individual factors, previous studies have introduced demographic characteristics and individual psychological perception into the study of safety voice behavior. There are few studies on individual characteristics such as ability and personality in the formation mechanism of safety voice behavior. The lack of studies in this area makes it challenging to integrate the current theoretical results into a systematic influence mechanism. Therefore, it is urgent to explore new variables to understand the mechanism of safety voice behavior fully. After the concept of political skill was proposed, it has received extensive attention from scholars in related fields. Its predictive effect on individual behavior in organizations has been confirmed by many theoretical studies. However, the research on whether political skill can affect employee safety voice behavior and its influence mechanism has not been reported yet. Based on the above analysis, this study connects political skill with safety voice behavior for the first time and examines how political skill affects safety voice behavior.

### 5.1. Political Skill and Safety Voice

The research first analyzed the motivation of employees’ safety voice behavior to explore the relationship between political skill and safety voice behavior. Voice is usually regarded as one of the vital impression management activities by employees in the organization [78]. Considering the positive effect of impression management behavior on career success [46], employees often adopt impression management strategies in the work environment to meet their career needs. Appropriate use of impression management strategies can establish a good personal image to gain the appreciation of leaders and colleagues and ultimately promote individual career success. In an organizational context, career success is usually pursued by employees, so they tend to actively take impression management strategies to achieve their expectations. This study proposes that the reasonable use of safety voice behavior and the proper presentation of safety issues to leaders and colleagues are effective means of impression management. Employees with political skill will regard safety voice behavior as a means of impression management, and the pursuit of career success will prompt them to produce safety voice behavior intentions.

From the results of hierarchical regression analysis, it can be seen that hypothesis H1 of this paper holds, that is, political skill can significantly promote workers’ safety voice behavior in high-risk industries. The ability of individuals with high political skill to flexibly adjust their own behavior according to the situation is observed in the eliciting of specific responses from others [64,67]. Therefore, when employees master high-level political skill, they will implement some behaviors conducive to the organization to create a good impression of their leaders so as to obtain rewards or promotion opportunities [79]. As safety voice plays a key role in reducing accidents, it may be considered by leaders as a behavior conducive to organizational development. Therefore, employees with strong political skill will use safety voice for the purpose of pleasing leaders. First, employees with higher political skill can often master how to use voice behavior as an effective strategy for impression management so that they can better exert the impression management effect of safety voice behavior. Individual political skill usually contributes to good social alertness [76]. Employees with strong political skill also have more precise control over when, where, and in what ways to use voice [47], which means that they are generally more likely to achieve the goal of safety voice behavior. Second, political skill can help reduce the risk of interpersonal conflict in the process of safety voice. Studies have suggested that voice behavior is accompanied by the risk of interpersonal conflict [62]. When making suggestions, a good foundation in interpersonal relationships may prevent leaders and colleagues from creating resistance and disgust. Individuals with high levels of political skill usually have excellent interpersonal influence and network skills [64]. Therefore, their suggestions are more likely to be valued and approved by leaders and colleagues. Additionally, the ideal effect of employee safety voice will drive them to make suggestions for the organization more actively, thereby forming a positive feedback causal chain that promotes the mutual promotion of employee safety voice and leaders’ high evaluation. In summary, employees with higher political skill can usually play a better impression management strategy when using safety voice.

### 5.2. The Mediating Role of Voice Efficacy

The empirical results of this study support Hypothesis H2, that is, political skill is positively correlated with voice efficacy. Political skill is necessary for survival in an organization and is closely related to employee rewards and organizational advancement [47]. Zellars et al. [80] found that employees with high political skill can flexibly adapt their behavior to the organization’s specific situation and have a higher sense of self-efficacy. The relationship between political skill and voice efficacy can be explained in two ways. On the one hand, employees with high political skill are at the key nodes of the organizational network and have good interpersonal resources [65]. Such resources are more likely to come from good relationships with supervisors and are important for others in the organization. Voice is risky, challenging, and can easily offend others [81]. Employees with interpersonal resources are confident that they can avoid interpersonal conflict by virtue of their good relationships with others [82,83]. Thus, a high level of political skill is accompanied by high perceptions of voice efficacy. On the other hand, employees with high skill levels are very responsive to the outside world [64]. They have the ability to grasp the timing and way of voice so as to achieve better voice effect. They are more confident that they will obtain good results from their voice, such as a good image in the organization, and this confidence is a reflection of their sense of voice efficacy.

We found that Hypothesis H3 holds and that voice efficacy has a positive predictive effect on safety voice. Safety voice behavior can have negative consequences for employees [10,84]. For example, leaders may perceive that employees are challenging the management status quo by raising safety issues and, therefore, become disgruntled and treat them harshly at work [14]. As a result, even if employees are motivated to voice safety concerns, there is a potential risk that they may eventually give up on the idea. Employees will only implement safety voice if they are confident that it will be effective. Employees with high self-efficacy are confident in their own abilities, actively engage with the organization, and exhibit more proactive behaviors [85]. Several studies have validated the role of self-efficacy in driving employees’ voice behavior [23,24]. Domain-specific self-efficacy has greater predictive power than general self-efficacy [54,86,87]. Voice efficacy is a proximal driver of safety voice. Employees with high levels of voice efficacy have the confidence to adapt their strategies for voice to organizational situations to achieve good voice outcomes [28]. Therefore, people with a high sense of voice efficacy are more likely to exhibit safety voice behavior.

In addition, our research results prove that voice efficacy plays a mediating role between political skill and safety voice. Hypothesis H4 holds. To clarify the mechanism of safety voice behavior, previous studies have introduced many intermediary variables into theoretical research models. It has been found that factors such as emotion-based trust [37] and perception of colleagues’ safety support [12] play an intermediary role in the influence mechanism of safety voice behavior. Previous studies mainly focused on selecting intermediary variables for cognitive factors. This may be because cognition often plays a key role in shaping individual behavior. It can be seen that using cognitive factors as a bridge connecting individual characteristics and behaviors can explain the relationship between variables more clearly. Before conducting safety voice, employees must have confidence in the effect of the behavior. Otherwise, they may give up safety voice and remain silent [55]. Voice efficacy is considered to be a construct closer to voice behavior. However, in the research of safety voice, there are few discussions about voice efficacy as a mediating variable.

The positive mediation effect of voice efficacy can be explained by two aspects. First, a high level of voice efficacy is beneficial to reducing employees’ risk perception of safety voice behavior. Noort et al. [44] verified the relationship between risk perception and safety voice behavior through experiments. Since political skill can serve to reduce the negative impact of a change-oriented voice on relationships [88], it also helps employees to increase their confidence in voice. Therefore, political skill can be regarded as a resource that helps employees better avoid the risk of interpersonal conflicts accompanying safety voice behavior, thereby enhancing their belief in raising workplace safety issues. Before employees make a safety voice behavior, they will make a lower assessment of the risk of the behavior, and they will more closely consider the positive feedback that the voice behavior may gain. In other words, due to the increase in the level of voice efficacy, the individual’s benefit evaluation of the voice behavior will take precedence over the risk perception of the behavior, so they will be more inclined to express their opinions. Second, since the voice of employees with high political skill is more likely to be adopted, individuals who have a successful voice experience will enhance their sense of voice efficacy, thus forming a good circle of positive promotion. In response to the voice made by employees, leaders will show selectivity when adopting it. On the one hand, political skill can not only help employees form a harmonious relationship between superiors and subordinates [89], but it can also improve the quality of the content of voice [81]. Moreover, voice efficacy makes employees more confident when raising safety-related issues [90], which helps leaders trust their professionalism in the safety-related work environment [91]. As a result, the possibility of voice being adopted also increases. On the other hand, voice efficacy is the key to determining whether employees will make safety voice. Those who believe that they can succeed in using voice tend to have higher intentions of safety voice behavior, so they are more willing to put forward their own opinions when facing safety issues in the workplace. In summary, considering the lower risk of interpersonal conflict and the higher possibility of adoption of voice, employees with high levels of political skill will have a more positive assessment of the effect of their suggestions, thereby enhancing the efficacy of individual voice. Therefore, employees are more convinced that they can play the role of the voice maker and ultimately implement safety voice behavior.

### 5.3. Theoretical Implications

The research results have some theoretical implications. Firstly, the study identified political skill as an important antecedent for employees to use safety voice in the organization actively. Safety voice contributes to the reduction of accident rates and is important for the organization’s development [10]. Employees with high levels of political skill are often willing to actively engage in safety voice for the sake of enhancing their image. Xue et al. [17] found that political skill can facilitate employees’ expression of opinions at work. This study confirms the positive relationship between political skill and safety voice, further corroborating ([17]) findings and enriching the theoretical model of the factors influencing safety voice behavior. While previous studies on individual factors influencing safety voice have mostly focused on demographic variables or variables that are closer to safety voice (e.g., organizational perceptions of safety support [92]), this paper introduces political skill, a variable that appears to be more distantly related to safety voice, to enrich the theoretical model of factors influencing safety voice. Although there is little research that directly explores the relationship between political skill and safety voice behavior, in the context of China’s ‘relational’ society, it can be assumed that people who can build a good image and avoid interpersonal conflict through safety voice behavior are more likely to offer advice to leaders. In addition, current research on safety voice has mainly focused on Western countries [10]. This study reveals the impact of this research on safety voice from the perspective of employees’ political skill in Chinese organizational contexts, adding to the existing research. Finally, scholars currently discuss how political skill contributes to employee behavior based on social exchange theory, resource conservation theory, and so on. This study takes the perspective of impression management motivation and argues that employees with strong political skill tend to have high impression management motivation and are more likely to build a good image of caring for the organization through safety voice, gaining the affection and admiration of their leaders and colleagues, and thus improving their organizational status. This study extends the research path of impression management theory to the context of safety voice behavior.

Secondly, the study identified a mediating role between political skill and safety voice behavior, with political skill facilitating safety voice behavior by increasing voice efficacy. This reveals a ‘black box’ in the relationship between perceived efficacy and safety voice. Although safety voice can contribute to improving organizational safety, it carries some risks [14]. For example, safety voice implies dissatisfaction with existing safety regulations and challenges the status quo, and employees may be deterred from making safety voice for these reasons. A sense of efficacy is an important psychological and cognitive condition in the decision-making process [93]. Employees with a high perception of efficacy have sufficient confidence to successfully use safety voice and obtain a good outcome. While past research has focused on the relationship between voice efficacy and voice, this paper demonstrates that voice efficacy positively influences safety voice and deepens the understanding of voice efficacy. Based on social cognitive theory, Thomas Taiyi Yan et al. [93] found that female leaders motivate employees to express work-related opinions more than male leaders in construct efficacy. This study also demonstrates that voice efficacy positively influences safety-specific voice from a social cognitive perspective, echoing a Thomas Taiyi Yan et al. [93] study, and it identifies a mediating role for voice efficacy in the relationship between political skill and safety constructs. The findings deepen the research on the relationship between voice efficacy and safety voice behavior and help construct the formation of safety voice behavior.

### 5.4. Practical Implications

The research results of this paper have important practical implications for enterprise safety management practices. First, the role of political skill on employee safety voice behavior should be taken seriously. Companies can organize learning or training to correctly understand political phenomena in the work environment and eliminate misunderstandings about political skill. In addition, organizations should regularly assess the level of political skill of employees, which allows employees to clearly understand their level of political skill. Organizations can also accurately grasp the political skill level of employees and make targeted improvement measures based on the feedback results.

Second, managers can enhance the employee’s voice efficacy to motivate employees to actively contribute to the organization’s safety issues. The influence of leaders and colleagues on individual safety voice behavior should be taken seriously by the organization. Leaders should give more support, encouragement, and praise to the safety voices of subordinates, encourage subordinates to actively put forward thoughts on safety issues, and shape the positive emotions of subordinates. Simultaneously, the organization should pay attention to the role model of the colleague’s successful voice experience on the individual. The superiors should give praise or material rewards to employees who actively contribute to the organization’s safety issues to stimulate the willingness of other employees to make safety voice.

### 5.5. Limitations and Future Research

(1) All variables in this study were measured using employee self-assessment, which may have resulted in a lack of objectivity in the study results. Therefore, to improve the accuracy of the variables measured, future studies may try to use a multi-source assessment approach to data collection, integrating information from different sources (e.g., employees’ leaders and colleagues), and thus produce more accurate evaluation results of the variables.

(2) In terms of research methods, although previous studies have obtained reasonable results using questionnaires, it is difficult for questionnaires to avoid influencing the research results because of the subjective thoughts of the research subjects. In addition to using questionnaires to study the mechanism of the occurrence of safety voice behavior in the future, research methods such as in-depth interviews and case studies can also be tried.

(3) In the course of this study, the collection of respondents in the questionnaire survey was mainly focused on the building construction industry, the hazardous chemicals industry, and the machinery manufacturing industry. The representativeness of the data is somewhat limited in terms of industry, which may lead to a lack of universality and representativeness of the sample, resulting in low generalization value of the research findings. Therefore, samples should be collected from different industries in future studies to expand the sample sources and enhance the universality and representativeness of the samples.

## 6. Conclusions

Based on impression management theory and social cognition theory, this study used the questionnaire survey method, processed and analyzed the collected data, and verified the positive correlation between political skill and safety voice behavior and the intermediary role of voice efficacy. The study results indicated that political skill positively affects safety voice, and a higher level of political skill drives employees to express safety-related suggestions to the organization. Additionally, we found that voice efficacy plays a mediating role in the model; that is, political skill affects safety voice behavior by acting on employees’ voice efficacy. In contrast to previous studies, which have mostly been based on other-oriented motivations for voice, we have introduced political skill as a variable for measuring resources in the study of safety voice. This study paid attention to safety voice, which is crucial to workplace safety, revealed the intermediary mechanism of political skill influencing safety voice, found the boundary conditions of political skill influencing safety voice through the sense of voice efficacy, and constructed the intermediary effect model, which is an important expansion of the research on antecedents of safety voice. This study enriches the theoretical and empirical findings in this area. The question of whether there are other ways of acting between political skill and safety voice has become an important direction for further research.

## Figures and Tables

**Table 1:** Reliability analysis.

Variable	Cronbach a Coefficient	Number of Items
Political skill	0.919	18
Voice efficacy	0.828	7
Safety voice behavior	0.814	5

**Table 2:** Confirmatory factor analysis results.

Model	$\chi^{2}/df$	RMR	RMSEA	CFI	IFI
Three-factor model	1.675	0.033	0.053	0.909	0.910
Two-factor model 1	1.921	0.038	0.061	0.875	0.876
Two-factor model 2	2.160	0.044	0.069	0.842	0.844
Two-factor model 3	1.835	0.037	0.058	0.887	0.888
Single factor model	2.271	0.044	0.072	0.827	0.829

Note: The three-factor model comprises political skill, voice efficacy, and safety voice behavior. Two-factor model 1 integrates political skill and voice efficacy into one variable. Two-factor model 2 integrates political skill and safety voice behavior into one variable. The two-factor model 3 integrates voice efficacy and safety voice behavior into one variable. The single-factor model is to integrate all variables into one variable.

**Table 3:** Correlation analysis results.

Variable	1	2	3	4	5	6	7	8
1	1							
2	0.01	1						
3	0.02	−0.17 **	1					
4	0.01	0.84 **	−0.20 **	1				
5	−0.11	0.16 *	0.10	0.21 **	1			
6	0.00	0.06	−0.06	0.06	0.01	1		
7	−0.13 *	0.10	−0.04	0.11	0.09	0.74 **	1	
8	−0.05	0.10	−0.03	0.08	0.07	0.56 **	0.66 **	1

Note: 1 is gender; 2 is age; 3 is education level; 4 is working years; 5 is position; 6 is political skill; 7 is voice efficacy; 8 is safety voice behavior; “**” means *p* < 0.01; “*” means *p* < 0.05.

**Table 4:** Hierarchical regression analysis results.

Regression Equation (N = 245)		Goodness of Fit Index			Significance of the Coefficient	
Predicted Variable	Predictor Variable	R	R^2^	F (df)	B	t
Safety voice		0.57	0.32	18.65 **		
	Gender				−0.06	−0.73
	Age				0.09	1.21
	Education				0.01	0.10
	Working years				−0.06	−0.65
	Position				0.05	0.92
	Political skill				0.70	10.31 **
Voice efficacy		0.75	0.57	51.58 **		
	Gender				−0.15	−2.92 *
	Age				−0.00	−0.09
	Education				0.01	0.34
	Working years				0.05	0.80
	Position				0.04	1.20
	Political skill				0.76	17.08 **
Safety voice		0.67	0.45	28.04 **		
	Gender				0.04	0.61
	Age				0.09	1.39
	Education				−0.00	−0.05
	Working years				−0.09	−1.12
	Position				0.02	0.42
	Voice efficacy				0.68	7.60 **
	Political skill				0.19	2.05 *

Note: “**” means *p* < 0.01; “*” means *p* < 0.05.

**Table 5:** Decomposition table of the total effect, direct effect, and intermediate effect.

	Effect	Standard Error	Boot LLCI	Boot ULCI	Relative Effect Value
Mediation effect	0.52	0.09	0.34	0.70	73.34%
Direct effect	0.19	0.11	−0.03	0.39	26.66%
Total effect	0.70	0.08	0.53	0.85	

## Data Availability

The data presented in this study are available on request from the corresponding author.
